# Supplementary material for: Anatomically and functionally distinct thalamocortical inputs to primary and secondary mouse whisker somatosensory cortices
Source: Nat Commun. 2020 Jul 3;11:3342. doi: 10.1038/s41467-020-17087-7 (PMC7335197; doi:10.1038/s41467-020-17087-7)
Supplement: Supplementary file 1 — Supplementary Information [file 41467_2020_17087_MOESM1_ESM.pdf]

Anatomically and functionally distinct thalamocortical inputs to primary and secondary mouse whisker somatosensory cortices

Sami El-Boustani, B. Semihcan Sermet, Georgios Foustoukos,

Tess B. Oram, Ofer Yizhar and Carl C.H. Petersen

SUPPLEMENTARY INFORMATION

Supplementary Figures 1-5

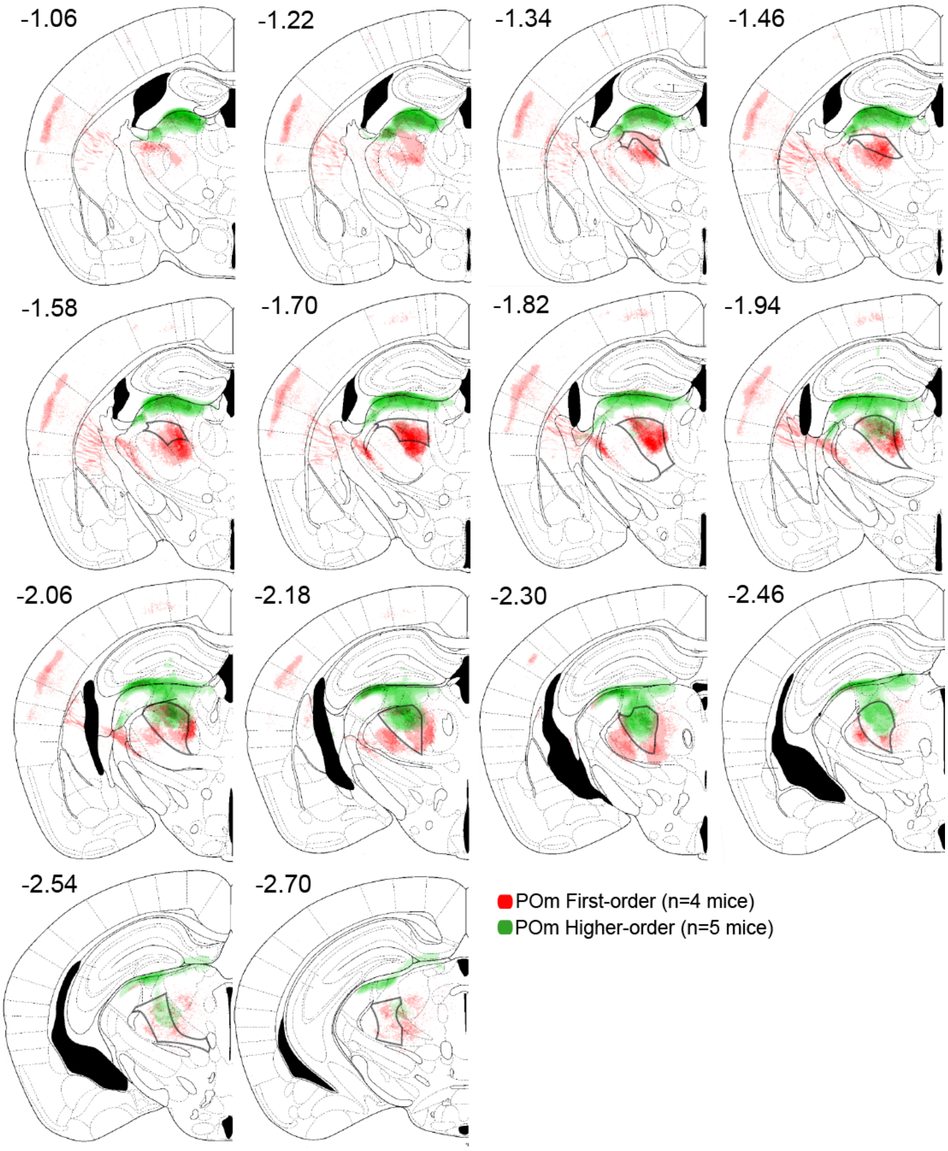

Supplementary Figure 1. Normalized fluorescent tracer expression domains relative to reference atlas[1] for POm First-order (red, n=4) and Higher-order (green, n=5) nuclei. Data for individual brains were acquired after transcardial perfusion either by imaging serial coronal brain slices mounted on slides or through serial two-photon tomography scanning of whole brains. Images of specific coronal slices were then scaled to fit the atlas for each mouse individually. The overall fluorescence for each mouse and channel was normalized by first subtracting the background fluorescence (95th percentile) as measured in the same thalamic region of the other hemisphere. We then saturated images to high percentile value (95th or 99th) so that the thalamic region with expression appeared homogeneously fluorescent. The fluorescent signal throughout the brain was then normalized to 1 for the maximum saturated value. Finally these normalized expression patterns were averaged across all mice. Bold outline: POm as defined in the reference atlas[1]. Note that expression of eYFP in neurons that are not expressing Cre-recombinase is not strictly limited to POm due to viral vector contaminating neighboring regions below the hippocampus such as the lateral posterior nucleus (LP) that is a higher-order thalamic nucleus part of the visual system.

The schematic drawings of the brain are reproduced from Paxinos & Franklin (2001) with permission from Elsevier.

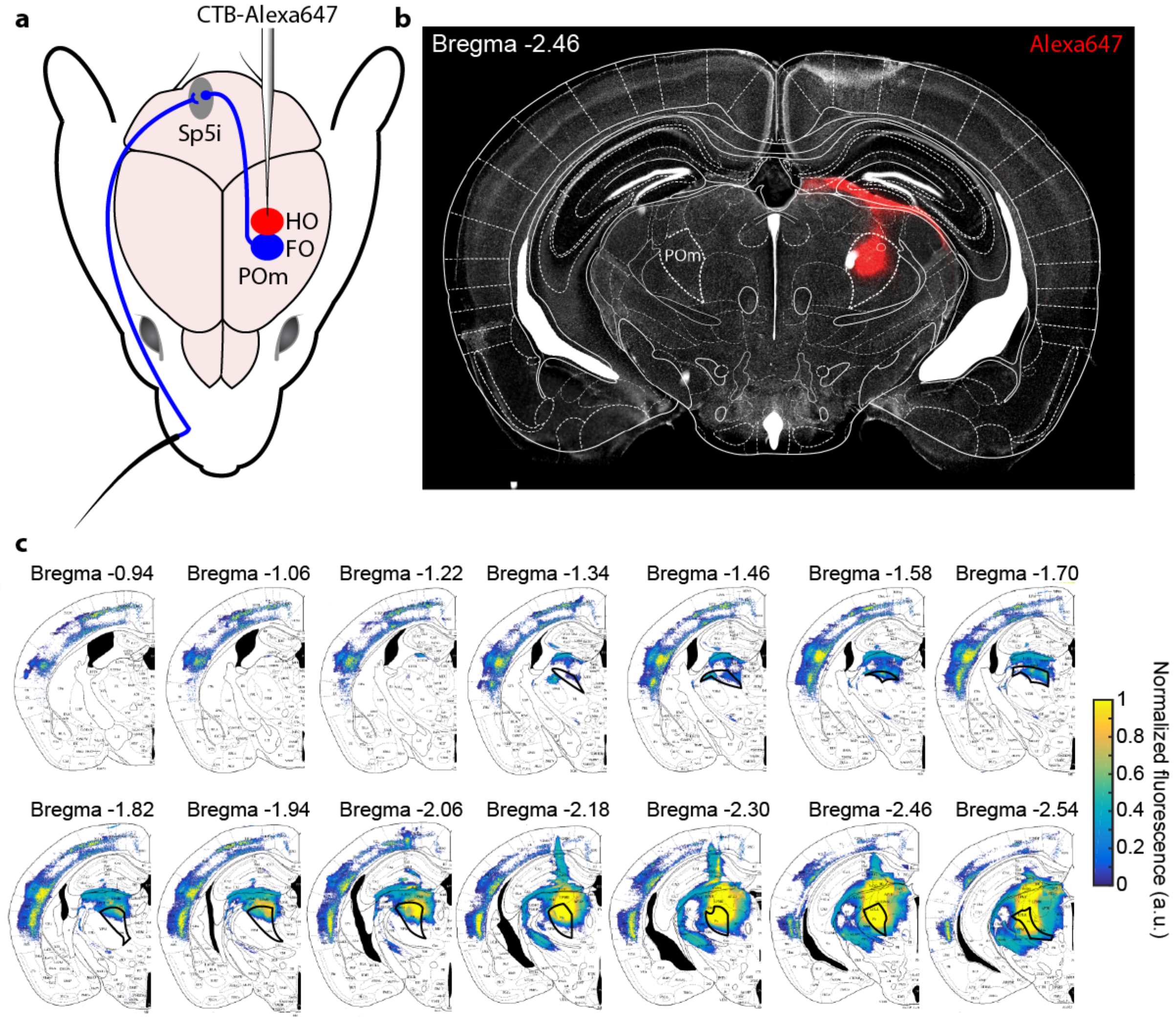

Supplementary Figure 2. POm higher-order nucleus receives cortical inputs from both layers 5 and 6. **a**, Injections of the cholera toxin subunit B (CTB) conjugated with Alexa647 were performed in the most posterior part of POm corresponding anatomically to the higher-order nucleus. **b**, Coronal brain slice after transcardial perfusion of these mice where the site of injection is visible in the POm-HO region. The image is aligned to the reference atlas[1]. **c**, For each mouse, we first aligned coronal brain slices to the reference atlas[1]. The fluorescent signal was then normalized to the maximum value throughout the cortex using a mask for each slice to only capture signal from POm-projecting neurons. These normalized brain maps were then averaged across mice (n=2 mice). Bold outline: POm as defined in the reference atlas[1].

The schematic drawings of the brain in panels b and c are reproduced from Paxinos & Franklin (2001) with permission from Elsevier.

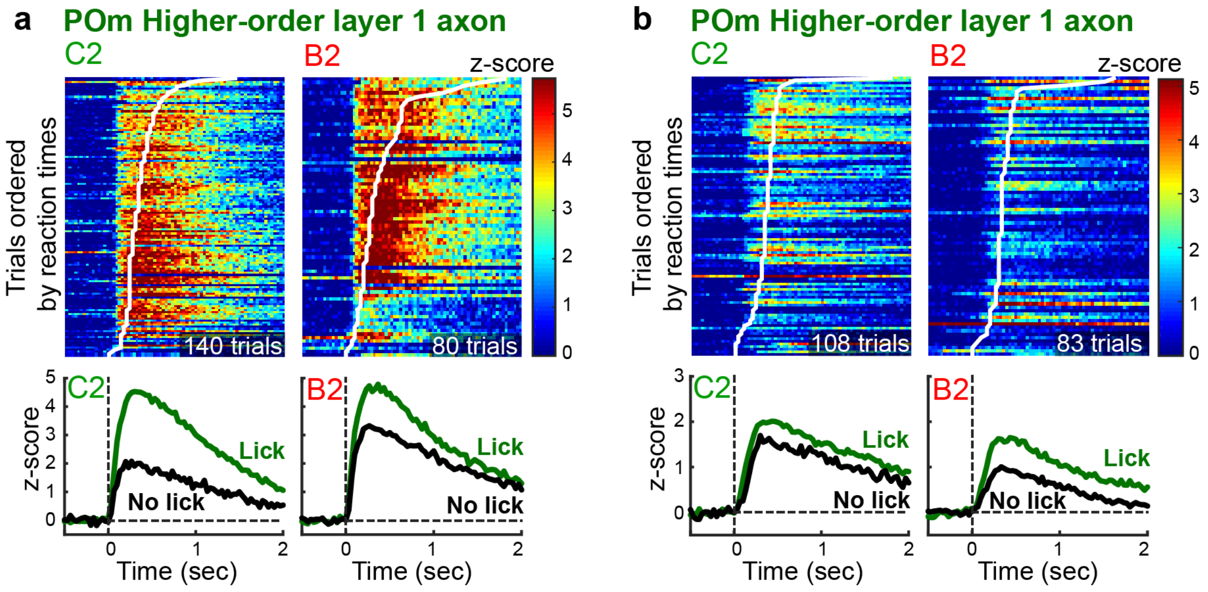

Supplementary Figure 3. Response examples of two POm-HO axons from layer 1 during lick trials. **a**, Calcium responses (z-score) for an example POm-HO axon during lick trials upon C2 or B2 whisker stimulation. Trials are ordered according to lick reaction times, which are shown with a white line on color maps. Average responses are shown below for lick (green) and no-lick conditions (black). **b**, same as **a** for another axon. Here we observe that some axons that were weakly tuned to a whisker would completely loose their selectivity as soon as the mouse licks in response to whisker stimulation. These responses during lick trials were amplified prior to the first lick reaction time as shown in these examples with no correlation with licking timing. We hypothesized that changes in selectivity could result from the overall increase in cortico-thalamic feedback during lick trials.

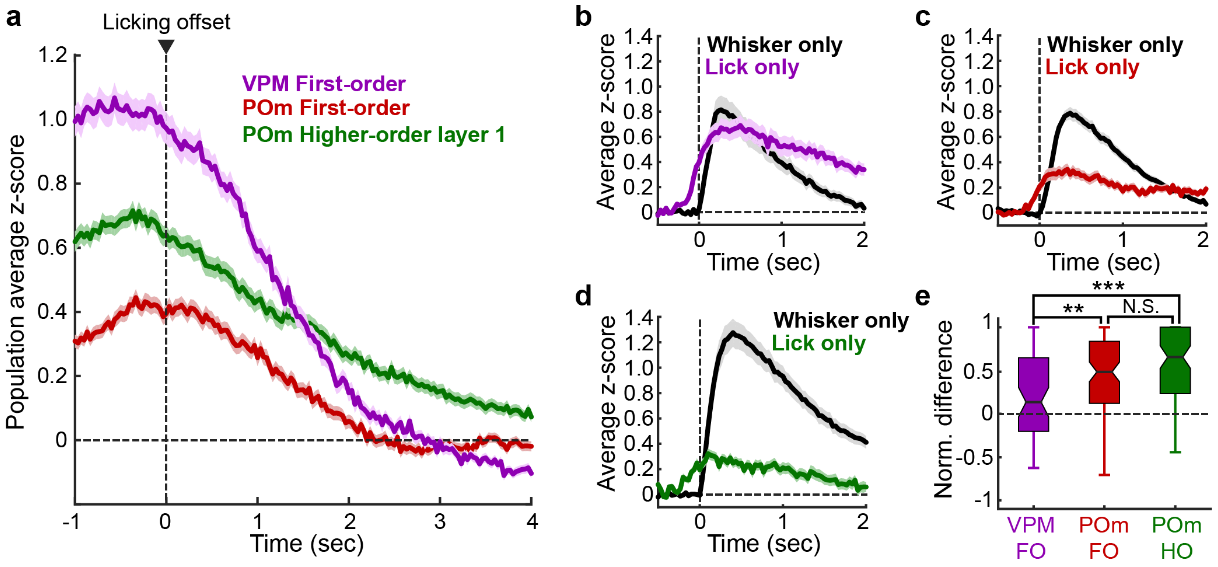

Supplementary Figure 4. Calcium responses measured at licking offset or during isolated lick events are stronger for VPM-FO axons. **a**, Population average z-score for all axons in each thalamo-cortical population around licking offset. For lick trials following C2 whisker stimulation, we identified the end of licking episode using face filming and aligned all calcium traces on this specific timing. All trials where mice licked again within 2 s following licking offset and trials with a very late licking offset close to the end of the trial (>5 s) were discarded. All axons with significant responses during passive whisker stimulation or with lick-related responses were included (n=139 axons for VPM-FO, n=235 axons for POm-FO, n=236 axons for POm-HO). **b-d**, For all axons with significant responses to passive whisker stimulation, we compared average z-score time course evoked by passive whisker stimulations (whisker only) to responses evoked during isolated lick events in absence of passive whisker stimulation (lick only). The origin 0 indicates either the stimulus onset or lick onset. **e,** Normalized differences between the two responses shown in **b-d** average over a window of 2s and compared across thalamocortical populations (n=62 axons for VPM-FO, n=97 axons for POm-FO, n=86 axons for POm-HO, ANOVA test, $p=6x10^{-5}$; Kruskal-Wallis two-sided test with Bonferroni correction, p=0.008 for VPM-FO vs POm-FO, $p=3x10^{-5}$ for VPM-FO vs POm-HO, p=0.3 N.S. not significant for POm-FO vs POm-HO). Boxplot: central mark indicates the median and edges indicate 25th and 75th percentiles. The whiskers extend to the largest or smallest point comprised within 1.5x of the interquartile range from both edges.

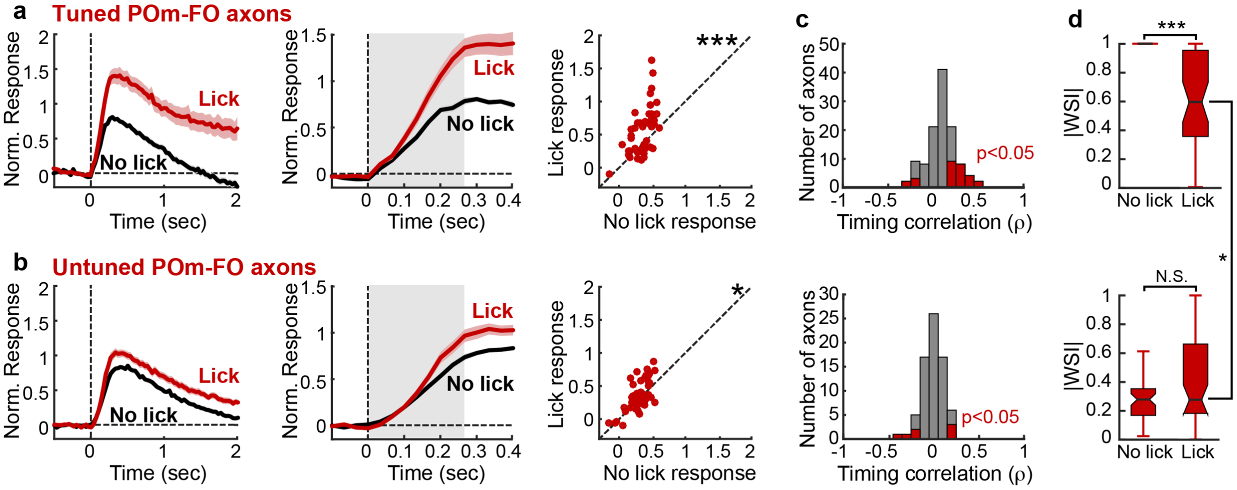

Supplementary Figure 5. Comparison of tuned (|WSI|>0.75) and untuned (|WSI|<0.75) POm-FO axons during goal-directed behavior. **a**, Left: Calcium responses averaged over all tuned POm-FO axons with significant responses to whisker stimuli during lick and no-lick trials, normalized to the no-lick condition. Dark lines: mean value and shaded areas: s.e.m. Middle: Early phase of the response over the first 0.4 sec. Right: Comparison of the response amplitude between lick and no-lick conditions averaged over the gray area (0 to 0.266 sec) in middle panel (n=46 axons, Wilcoxon paired two-sided test, ***p=4x10^{-5}). **b**, Same as **a**, but for untuned POm-FO (n=53 axons, Wilcoxon paired two-sided test,*p=0.01). **c**, Distributions of Pearson correlation coefficient between reaction times and calcium response latencies for all axons with significant responses in lick trials (n=116 for tuned POm-FO axons, n=73 for untuned POm-FO axons). Colored bars: Pearson coefficient with p<0.05. **d**, Distributions of whisker selectivity index absolute values comparing tuned and untuned POm-FO axonal populations and lick/no-lick conditions for axons with significant sensory responses in no-lick condition (Kruskal-Wallis two-sided test with Bonferroni correction; tuned POm-FO: ***p=3x10^{-6} for lick vs no-lick, POm-FO: p=1 for lick vs no-lick N.S. not significant, *p=0.013 for tuned POm-FO vs untuned POm-FO in lick condition). Boxplot: central mark indicates the median and edges indicate 25th and 75th percentiles. The whiskers extend to the largest or smallest point comprised within 1.5x of the interquartile range from both edges.

Supplementary References

1. Paxinos, G. & Franklin, K. B. J. *The Mouse Brain in Stereotaxic Coordinates*. (Academic Press Inc, 2001).
