# Supplementary material for: Anatomically and functionally distinct thalamocortical inputs to primary and secondary mouse whisker somatosensory cortices
Source: Nat Commun. 2020 Jul 3;11:3342. doi: 10.1038/s41467-020-17087-7 (PMC7335197; doi:10.1038/s41467-020-17087-7)
Supplement: Supplementary file 3 — Reporting Summary [file 41467_2020_17087_MOESM3_ESM.pdf]

nature research

Corresponding author(s): Sami El-Boustani & Carl C.H. Petersen

Last updated by author(s): May 21, 2020

Reporting Summary

Nature Research wishes to improve the reproducibility of the work that we publish. This form provides structure for consistency and transparency in reporting. For further information on Nature Research policies, see Authors & Referees and the Editorial Policy Checklist .

Statistics

For all statistical analyses, confirm that the following items are present in the figure legend, table legend, main text, or Methods section.

n/a	Confirmed	
☐	☒	The exact sample size (n) for each experimental group/condition, given as a discrete number and unit of measurement
☐	☒	A statement on whether measurements were taken from distinct samples or whether the same sample was measured repeatedly
☐	☒	The statistical test(s) used AND whether they are one- or two-sided<br>*Only common tests should be described solely by name; describe more complex techniques in the Methods section.*
☒	☐	A description of all covariates tested
☐	☒	A description of any assumptions or corrections, such as tests of normality and adjustment for multiple comparisons
☐	☒	A full description of the statistical parameters including central tendency (e.g. means) or other basic estimates (e.g. regression coefficient) AND variation (e.g. standard deviation) or associated estimates of uncertainty (e.g. confidence intervals)
☐	☒	For null hypothesis testing, the test statistic (e.g. F, t, r) with confidence intervals, effect sizes, degrees of freedom and P value noted<br>*Give P values as exact values whenever suitable.*
☒	☐	For Bayesian analysis, information on the choice of priors and Markov chain Monte Carlo settings
☒	☐	For hierarchical and complex designs, identification of the appropriate level for tests and full reporting of outcomes
☐	☒	Estimates of effect sizes (e.g. Cohen's d, Pearson's r), indicating how they were calculated

Our web collection on statistics for biologists contains articles on many of the points above.

Software and code

Policy information about availability of computer code

Data collection	Data collection was performed using the Matlab-based scanning software Scanimage 5.0 (Vidrio Technologies) and custom-made code for behavior and intrinsic optical imaging running on Matlab 2015a (Mathworks). In vitro patch experiments were performed using Igor Pro 6.21 (WaveMetrics). Post-mortem imaging of brain slices were performed with Leica software Las X or with Scanimage 5 for the serial tomography. For the two-photon serial-section microscopy, we also used the Matlab-based software BakingTray.
Data analysis	All analysis were performed using Matlab 2015a or 2018 (Mathworks). Processing of in vivo calcium imaging data was first done with the Matlab-based software Suite 2p. For the two-photon serial-section microscopy, processing was done with the Matlab-based softwares StitchIt and MaSIV.

For manuscripts utilizing custom algorithms or software that are central to the research but not yet described in published literature, software must be made available to editors/reviewers. We strongly encourage code deposition in a community repository (e.g. GitHub). See the Nature Research guidelines for submitting code & software for further information.

Data

Policy information about availability of data

All manuscripts must include a data availability statement. This statement should provide the following information, where applicable:

- Accession codes, unique identifiers, or web links for publicly available datasets
- A list of figures that have associated raw data
- A description of any restrictions on data availability

The data and Matlab code used to generate figures that support the findings of this study are freely available in the Open Access CERN database Zenodo: https://zenodo.org/communities/petersen-lab-data with doi hyperlink: https://doi.org/10.5281/zenodo.3824359.

Field-specific reporting

Please select the one below that is the best fit for your research. If you are not sure, read the appropriate sections before making your selection.

- [x] Life sciences
- [ ] Behavioural & social sciences
- [ ] Ecological, evolutionary & environmental sciences

For a reference copy of the document with all sections, see nature.com/documents/nr-reporting-summary-flat.pdf

Life sciences study design

All studies must disclose on these points even when the disclosure is negative.

Sample size

For each experiment, enough data was collected to guarantee the validity of our statistical test. For most comparison a large number of axons could be imaged and therefore was sufficient to compare different experimental groups. For anatomical description, given the strong qualitative difference between our experimental groups (low variability within each group), we estimated that at least 4 mice with successful targeted expression were enough. Variances were computed for all groups and were generally in the same order of magnitude. No additional sample size calculation was performed as we could collect large n numbers for statistical tests performed.

Data exclusions

Mice for which the cranial window and prism surgery did not succeed (e.g. blood under the window) were excluded from analysis. For the patch experiments, we excluded neurons that displayed abnormal ChR2-evoked depolarization (>20mV which is not physiological) or that display abnormal resting potential indicating a bad patch configuration. For these same experiments, slice where weak or absent input was detected in input layers were discarded because they indicated a failure in the ChR2 expression (or inaccurate injection). In the anatomical description (Fig. 1 and in particular Supplementary Fig. 2), we discarded brains where post-hoc analysis revealed that the viral vector was targeted to the wrong region. As these exclusion criteria directly indicated a failure of the experiment, they were pre-established.

Replication

For all experiments, we followed rigorously the same procedures and kept track of all our steps in an electronic lab notebook. Some of the experiments presented here (e.g.: behavioral training) were replicated by other lab members producing similar results. These specific experiments were repeated four times independently and over several mice (between 2 and 5) in each experimental run. Successful overall training of these mice were observed systematically.

Randomization

No randomization of samples was done in any of our analyses. This was not relevant in this study since all groups received exactly the same treatment and in vivo and post-hoc analysis could confirm that we were targeting the right thalamic population.

Blinding

No blinding of samples was done in any of our analyses. This was not relevant in this study since all groups received exactly the same treatment and in vivo and post-hoc analysis could confirm that we were targeting the right thalamic population.

Reporting for specific materials, systems and methods

We require information from authors about some types of materials, experimental systems and methods used in many studies. Here, indicate whether each material, system or method listed is relevant to your study. If you are not sure if a list item applies to your research, read the appropriate section before selecting a response.

Materials & experimental systems

n/a	Involved in the study	
[ ]	[x]	Antibodies
[x]	[ ]	Eukaryotic cell lines
[x]	[ ]	Palaeontology
[ ]	[x]	Animals and other organisms
[x]	[ ]	Human research participants
[x]	[ ]	Clinical data

Methods

n/a	Involved in the study	
[x]	[ ]	ChIP-seq
[x]	[ ]	Flow cytometry
[x]	[ ]	MRI-based neuroimaging

Antibodies

Antibodies used

we used primary anti-GFP antibody (rabbit polyclonal 1:5000, Abcam 290, UK) and the secondary antibody (goat anti-rabbit conjugated to Alexa 488 1:200, Life Technologies A-11012)

Validation

Antibodies were used to amplify a pre-existing weak signal in fixed slices (eYFP). We observed fluorescence before and after the amplification which was the same only with a strong change in intensity.

Animals and other organisms

Policy information about studies involving animals; ARRIVE guidelines recommended for reporting animal research

Laboratory animals	C57BL/6 wild-type mice and heterozygote Gpr26-Cre mice54 (Tg(Gpr26-cre)KO250Gsat, JAX mouse number (4847098) were used in this study. For all experiments, we used adult mice from both sexes and aged between P25 and P300. They were housed in cages containing 1-5 mice under a 12/12-hour reverse light cycle. The ambient temperature in the animal facility was 23 °C and the relative humidity was maintained around 50%.
Wild animals	No wild animals were used in the study.
Field-collected samples	No field-collected samples were used in the study.
Ethics oversight	Experiments were carried out in mice under protocols approved by the Swiss Federal Veterinary Office (License number VD1628) and were conducted in accordance with the Swiss guidelines for the use of research animals.

Note that full information on the approval of the study protocol must also be provided in the manuscript.
