# Supplementary material for: Anatomically and functionally distinct thalamocortical inputs to primary and secondary mouse whisker somatosensory cortices
Source: Nat Commun. 2020 Jul 3;11:3342. doi: 10.1038/s41467-020-17087-7 (PMC7335197; doi:10.1038/s41467-020-17087-7)
Supplement: Supplementary file 4 — Description of Additional Supplementary Files [file 41467_2020_17087_MOESM4_ESM.pdf]

**Description of Additional Supplementary Files**

File Name: Supplementary Movie 1
Description: Serial two-photon scanning of a perfused brain expressing tdTomato in POm first-order neurons and eYFP in POm higher-order. The movie goes back and forth along the rostro-caudal axis to highlight the location of the two nuclei.

File Name: Supplementary Movie 2
Description: Two-photon z-stack imaging of VPM first-order axons in wS2 seen through a microprism in an awake mouse.
